# Supplementary material for: Assessing Temperature-Dependent Deltamethrin Toxicity in Various kdr Genotypes of Aedes aegypti Mosquitoes
Source: Insects. 2025 Mar 1;16(3):254. doi: 10.3390/insects16030254 (PMC11943331; doi:10.3390/insects16030254)
Supplement: Supplementary file 1 [file insects-16-00254-s001.zip › insects-3408175-supplementary.pdf]

Supplementary Information

Table S1 - Deltamethrin concentrations and solvent volumes. Composition of impregnated 12 x 15cm Whatman No. 1 papers for WHO tube assays, including the weight of technical grade deltamethrin (mg), acetone (ml), and olive oil (ml) at all target concentrations. A stock solution of deltamethrin in acetone was prepared and diluted serially to achieve the desired concentrations before mixing with olive oil. All volumes were converted to weight according to density, and concentrations were prepared gravimetrically.

Concentration (%)	Deltamethrin (mg)	Acetone (ml)	Olive oil (ml)
Control	-	2.0	0.704
0.001	0.007	2.0	0.704
0.003	0.021	2.0	0.704
0.005	0.036	2.0	0.704
0.006	0.043	2.0	0.704
0.01	0.071	2.0	0.704
0.02	0.142	2.0	0.704
0.03	0.214	2.0	0.704
0.05	0.356	2.0	0.704
0.07	0.498	2.0	0.704
0.10	0.712	2.0	0.704
0.20	1.424	2.0	0.704
0.25	1.780	2.0	0.704
0.30	2.136	2.0	0.704
0.50	3.560	2.0	0.704
0.70	4.985	2.0	0.704
1.0	7.121	2.0	0.704
2.0	14.24	2.0	0.704
3.0	21.36	2.0	0.704
4.0	28.48	2.0	0.704
5.0	35.60	2.0	0.704
7.0	49.85	2.0	0.704
